# Supplementary material for: Mechanisms governing the pioneering and redistribution capabilities of the non-classical pioneer PU.1
Source: Nat Commun. 2020 Jan 21;11:402. doi: 10.1038/s41467-019-13960-2 (PMC6972792; doi:10.1038/s41467-019-13960-2)
Supplement: Supplementary file 7 — Source data [file 41467_2019_13960_MOESM7_ESM.zip › Source_Data/FigureS2/FigureS2B_MotifScanOutput/KmeansCluster6/homerResults/motif15.similar.html]

motif15

## Information for motif15

C
G
T
A
A
G
C
T
A
C
G
T
C
T
A
G
C
T
A
G
T
C
G
A
G
C
A
T
T
A
G
C
T
C
G
A
A
G
C
T
C
T
A
G
A
C
T
G
T
C
A
G
C
A
T
G
  
Reverse Opposite:  

G
T
A
C
A
G
T
C
A
G
T
C
A
G
T
C
C
T
G
A
A
G
C
T
A
C
T
G
C
G
T
A
A
G
C
T
A
G
T
C
A
G
T
C
C
G
T
A
T
C
G
A
C
G
A
T
  

|  |  |
| --- | --- |
| p-value: | 1e-62 |
| log p-value: | -1.439e+02 |
| Information Content per bp: | 1.757 |
| Number of Target Sequences with motif | 124.0 |
| Percentage of Target Sequences with motif | 3.77% |
| Number of Background Sequences with motif | 172.1 |
| Percentage of Background Sequences with motif | 0.40% |
| Average Position of motif in Targets | 104.4 +/- 73.9bp |
| Average Position of motif in Background | 92.8 +/- 67.4bp |
| Strand Bias (log2 ratio + to - strand density) | -0.0 |
| Multiplicity (# of sites on avg that occur together) | 1.00 |
| Motif File: | file (matrix) reverse opposite |

### Similar de novo motifs found

|  |  |  |  |  |  |  |  |
| --- | --- | --- | --- | --- | --- | --- | --- |
| Rank | Match Score | Redundant Motif | P-value | log P-value | % of Targets | % of Background | Motif file |
| 1 | 0.969 | C G T A A G C T A C G T C T A G C T A G C G T A A C G T A G T C C G T A A C G T A C T G A C T G A C T G | 1e-58 | -135.303787 | 3.46% | 0.35% | motif file (matrix) |
| 2 | 0.906 | G A T C A G T C T C G A G C A T A T C G C G T A C G A T A G C T G A T C G T C A C T G A C G A T | 1e-54 | -124.549304 | 3.04% | 0.28% | motif file (matrix) |
| 3 | 0.887 | C G A T C T A G T C A G T C G A A G C T T A G C C G T A A C G T C A T G A C T G T A C G C T A G T C A G | 1e-45 | -104.172374 | 2.61% | 0.26% | motif file (matrix) |
| 4 | 0.751 | A G T C G A T C T G A C G A T C G A T C C G T A G A C T T A C G T G C A A G C T | 1e-43 | -99.971271 | 7.29% | 2.43% | motif file (matrix) |
| 5 | 0.890 | A C G T C T A G C T A G C G T A A C G T T G A C T C G A G A C T C T A G C A T G C T A G | 1e-42 | -98.863724 | 3.13% | 0.45% | motif file (matrix) |
| 6 | 0.781 | C T A G T A C G C T G A C A G T A T G C G T C A A C G T A C T G C T A G A C T G C T A G A C T G | 1e-40 | -93.183361 | 2.46% | 0.27% | motif file (matrix) |
| 7 | 0.813 | A G T C A T G C A G T C A G T C C T G A A G C T A C T G C G T A A C G T | 1e-35 | -81.173194 | 2.82% | 0.47% | motif file (matrix) |
